# A Concise Review of Pelvic Radiation Therapy (RT) for Rectal Cancer with Synchronous Liver Metastases

**DOI:** 10.1155/2019/5239042

**Published:** 2019-04-21

**Authors:** Omer Sager, Ferrat Dincoglan, Selcuk Demiral, Bora Uysal, Hakan Gamsiz, Bahar Dirican, Murat Beyzadeoglu

**Affiliations:** Department of Radiation Oncology, University of Health Sciences, Gulhane Medical Faculty, Ankara, Turkey

## Abstract

**Background and Objective:**

Colorectal cancer is a major health concern as a very common cancer and a leading cause of cancer-related mortality worldwide. The liver is a very common site of metastatic spread for colorectal cancers, and, while nearly half of the patients develop metastases during the course of their disease, synchronous liver metastases are detected in 15% to 25% of cases. There is no standardized treatment in this setting and no consensus exists on optimal sequencing of multimodality management for rectal cancer with synchronous liver metastases.

**Methods:**

Herein, we review the use of pelvic radiation therapy (RT) as part of potentially curative or palliative management of rectal cancer with synchronous liver metastases.

**Results:**

There is accumulating evidence on the utility of pelvic RT for facilitating subsequent surgery, improving local tumor control, and achieving palliation of symptoms in patients with stage IV rectal cancer. Introduction of superior imaging capabilities and contemporary RT approaches such as Intensity Modulated Radiation Therapy (IMRT) and Image Guided Radiation Therapy (IGRT) offer improved precision and toxicity profile of radiation delivery in the modern era.

**Conclusion:**

Even in the setting of stage IV rectal cancer with synchronous liver metastases, there may be potential for extended survival and cure by aggressive management of primary tumor and metastases in selected patients. Despite lack of consensus on sequencing of treatment modalities, pelvic RT may serve as a critical component of multidisciplinary management. Resectability of primary rectal tumor and liver metastases, patient preferences, comorbidities, symptomatology, and logistical issues should be thoroughly considered in decision making for optimal management of patients.

## 1. Background and Introduction

Colorectal cancer is a major health concern as a very common cancer and a leading cause of cancer-related mortality worldwide [1–4]. Cancers of the rectum account for a considerable proportion of all large intestine cancers and are typically included within the group of colorectal cancers in epidemiological studies [5]. The liver is a very common site of metastatic spread for colorectal cancers, and, while nearly half of the patients develop metastases during the course of their disease, synchronous liver metastases are detected in 15% to 25% of cases [6–12]. With ever-increasing advances in both surgical and clinical oncology disciplines, multimodality management has substantially improved outcomes of rectal cancer with oligometastases. If feasible, surgical removal of the primary rectal tumor along with resection/ablation of liver metastases as a potentially curative therapeutic approach has been shown to achieve survival rates exceeding 70% at 5 years for selected patients with limited disease burden [13–47].

The role of radiation therapy (RT) in the management of nonmetastatic locally advanced rectal cancers is well established; however, its utility in the setting of synchronous liver metastases needs elucidation. Herein, we address the utility of pelvic RT as part of potentially curative or palliative management of rectal cancer with synchronous liver metastases in light of the literature.

## 2. Rationale for Pelvic RT in the Setting of Rectal Cancer with Liver Metastases

The utility of RT has been well established in nonmetastatic locally advanced rectal cancer, and preoperative sequencing of pelvic irradiation rather than postoperative administration has been shown to reduce the risk of local recurrence and cancer related mortality [48–50]. In terms of dose and fractionation for preoperative pelvic irradiation, 2 main RT schemes include short course RT (SCRT) to deliver a total radiation dose of 25 Gy with 5 daily treatment fractions of 5 Gy each over 1 week and long course RT (LCRT) to deliver a total radiation dose of 45-50.4 Gy (with or without an additional boost of 5.4 Gy delivered in 3 fractions if circumferential resection margin is threatened) over 5 to 6 weeks with conventional fractionation (1.8-2 Gy per each fraction). While assessment of local failure, disease free survival (DFS), overall survival (OS), sphincter preservation, late toxicity, and quality of life revealed comparable outcomes with both preoperative RT approaches in several studies, practice patterns vary widely around the globe [51–58]. Surgery with total mesorectal excision (TME) has been traditionally scheduled within 1 week (immediate surgery) or 6 to 8 weeks after completion of SCRT or LCRT, respectively [48, 49, 59]. However, considering the increased pathological response rates achieved with a longer time interval between preoperative RT and surgery, delayed rather than immediate surgery after preoperative SCRT has been suggested as a viable therapeutic approach to improve treatment outcomes [60–69]. While incorporation of RT in multimodality treatment of nonmetastatic rectal cancers is widely accepted, its utility for management of rectal cancer is debated in the setting of synchronously detected liver metastases.

Given the increased life expectancy of patients with metastatic stage IV rectal cancer treated in the modern era using more effective local and systemic therapies, addressing of the primary disease becomes more critical to improve patient comfort and quality of life with contemporary RT techniques allowing improved toxicity profile. Indeed, 7% of the study population consisted of patients with stage IV rectal cancer in the landmark Dutch trial assessing preoperative RT followed by TME, and reported rates of 2-year local recurrence were 10.1% and 23.8% for patients treated with or without preoperative RT, respectively [70]. Other studies focusing on management of stage IV rectal cancer have also incorporated RT as part of multimodality management with several purposes including symptomatic palliation of symptoms, local control of primary disease, and facilitating subsequent TME surgery [71–87].

Several studies investigating the omission of RT in the setting of nonmetastatic or metastatic disease consistently reported inferior outcomes, substantiating the utility of RT in multimodality management of rectal cancer [88–91]. Moreover, in the setting of metastatic rectal cancer, improved primary tumor control by use of local therapy has been associated with improved prognosis and survival as well [92, 93].

## 3. Review of Studies Including Pelvic RT with Palliative Intent

Selected series incorporating pelvic RT in multimodality management of stage IV rectal cancer with liver metastases (with or without other distant metastases) as a palliative therapeutic strategy are summarized in Table 1.

Pelvic RT achieves effective palliation of symptoms due to primary tumor in stage IV rectal cancer. Two reviews focusing on studies conducted in different time periods (1949-1999 and 2011-2016) confirmed the palliative efficacy of RT for management of primary rectal tumor related symptoms [94, 95].

In the systematic review by Cameron et al. based on 27 studies, pooled overall symptomatic response rate was 75%, while response rates were 78%, 81%, 71%, and 72% for symptoms of pain, bleeding and discharge, mass effect, and other pelvic symptoms, respectively [94].

Buwenge et al. reviewed more recently published series and reported response rates of 79%, 87%, and 78% for symptoms of pain, bleeding, and obstruction, respectively [95]. In addition to 2 retrospective and 2 prospective series in our review (Table 1), these 2 reviews with different study periods revealed effective palliation of pelvic symptoms by using RT [94, 95]. Similar response rates in both earlier and more recent RT series suggest that the palliative efficacy of RT may not be neglected in the modern era and persists despite the availability of newer effective systemic treatments.

## 4. Pelvic RT as Part of Potentially Curative Multimodality Management of Rectal Cancer with Synchronously Detected Liver Metastases

Synchronous liver metastases have been variably defined in the literature. Most common definition includes metastases detected at or before diagnosis of primary rectal cancer; however, metastases detected within 3 to 6 months of diagnosis have also been included in the “synchronous” group in several studies [96–101]. Compared to metachronous liver metastases, synchronously detected liver metastases may be associated with poorer prognosis and survival [96, 102–104]. Even in this setting, there remains the potential for cure with multimodality management. Outcomes of selected series incorporating RT in multimodality management of rectal cancer with synchronously detected liver metastases are summarized in Table 2.

Despite heterogeneity in patient and treatment characteristics in these studies, several conclusions may be drawn.

Although with a very limited sample size, the study by Shin et al. using SCRT in multimodality management reported a high rate (84%) of R0 resection with no LR during the follow-up period [73].

In the study by Fossum et al. including 93 patients, LR was not observed in patients receiving neoadjuvant RT, and omission of neoadjuvant RT was associated with development of subsequent LR [91].

Overall, outcomes of 8 retrospective and 4 prospective studies reveal that pelvic RT may serve as a critical component of multidisciplinary management (Table 2). Contemporary RT techniques including Intensity Modulated Radiation Therapy (IMRT) and Image Guided Radiation Therapy (IGRT) along with timely management of radiation induced toxicity by use of nutritional supplementation may improve patient compliance and quality of life [106].

## 5. Conclusion

Even in the setting of stage IV rectal cancer with synchronous liver metastases, there may be potential for extended survival and cure by aggressive management of primary tumor and metastases in selected patients. Despite lack of consensus on sequencing of treatment modalities, pelvic RT may serve as a critical component of multidisciplinary management. Resectability of primary rectal tumor and liver metastases, patient preferences, comorbidities, symptomatology, and logistical issues should be thoroughly considered in decision making for optimal management of patients. Similar response rates in earlier and more recent palliative RT series suggest that the palliative efficacy of RT may not be neglected in the modern era and persists despite the availability of newer effective systemic treatments.

Given the increased life expectancy of patients with rectal cancer and synchronous liver metastases, addressing of the primary tumor with pelvic RT may have utility as part of potentially curative management. SCRT may be preferred in this setting to avoid delaying of systemic treatment, and improving distant as well as local control may be attempted by use of intensified chemoradiotherapy regimens. Modern RT techniques offer excellent precision and accuracy with an improved toxicity profile.

Backdating of liver surgery to be performed in the interval between RT and rectal surgery may be a viable therapeutic approach to shorten the overall treatment time and liver first strategies may be thoroughly investigated to avoid progression of liver metastases during the disease course. Intensification of systemic treatment with or without biological agents, immunotherapy, optimal dose and fractionation for RT, optimal sequencing, and combination of treatment modalities warrant further extensive research.

## Figures and Tables

**Table 1:** Selected series incorporating pelvic RT in multimodality management of stage IV rectal cancer with liver metastases (with or without other distant metastases) as a palliative therapeutic strategy.

Authors (Reference)	Study year	Study design	Number of patients	Disease status	RT dose and fractionation	Median follow-up (range)	Response to palliative RT

Crane et al. [85]	2001	Retrospective	80	Stage IV rectal cancer with synchronous distant metastases	30 Gy/6 fx (50%) 45 Gy/25 fx (16%) 35 Gy/14 fx (14%) Other (20%)	52 (3-444) weeks	Symptoms due to rectal tumor resolved in 94% Overall 1-year actuarial symptom control rate 85% Overall 2-year actuarial symptom control rate 82%
Bae et al. [86]	2011	Retrospective	80	Metastatic stage IV colorectal cancer	Median total RT dose 36 Gy (range: 8-60 Gy) Median dose per fraction 2.5 Gy (range: 1.8-8 Gy)	5 (1-44) months	Overall symptomatic palliation rate 80% Median symptom control duration 5 months (range: 1-44 months)
Tyc-Szczepaniak et al. [87]	2013	Prospective phase II study	40	Stage IV rectal cancer with synchronous distant metastases	25 Gy/5 fx (97.5%) 30 Gy/6 fx (2.5%)	26 (19-34) months	Complete resolution of pelvic symptoms during the whole course of disease in 12 patients (30%) Significant improvement in 14 patients (35%) 67% probability of a sustained good palliative effect at 2 years
Picardi et al. [72]	2016	Prospective phase II study	18	Stage IV rectal cancer with synchronous distant metastases	25 Gy/5 fx	11.5 (3-36) months	Complete response (i.e., complete resolution of symptoms) at 4 weeks after treatment in 7 patients (38.9%) Partial response at 4 weeks after treatment in 9 patients (50%) Reduction or resolution of pain 87.5% Reduction or resolution of bleeding 100%

**Table 2:** Selected series incorporating pelvic RT as part of potentially curative multimodality management of rectal cancer with synchronously detected liver metastases (with or without other distant metastases).

Authors (Reference)	Study year	Study design	Number of patients	Disease status	RT dose and fractionation	Median follow-up (range)	Response to RT
Shin et al. [73]	2011	Retrospective	6	Stage IV LARC with synchronous distant metastases	25 Gy/5 fx	16.7 (15.5-23.5) months	No local recurrence during follow-up period R0 resection accomplished in 5 patients (84%)
Salgado et al. [74]	2014	Retrospective	26	Stage IV LARC with synchronous distant metastases	45 ±5.4 Gy/25-28 fx	-	1-year OS 95% 2-year OS 70% Mean survival time 40.5 months 1-year PFS 91% 2-year PFS 36% Mean PFS time 23.1 months 1-year LC 91% 2-year LC 66%
Yoon et al. [83]	2016	Retrospective	50	Stage IV LARC with synchronous distant metastases	25 Gy/5 fx	22 (9-59) months	Median PFS 16 months 2-year PFS 34.8% 2-year OS 73.9% 5-year OS 55.1%
Cho et al. [82]	2016	Prospective multicenter randomized phase 2 study	38	Stage IV rectal cancer with synchronous liver metastases	45 ±5.4 Gy/25-28 fx	-	Overall R0 resection rate for both the primary tumor and liver metastases was 77.8% in arm A (induction CapeOx + CapeOx-RT) and 70% in arm B (CapeOx-RT alone) Median PFS 14.2 months in arm A and 15.1 months in arm B 3-year OS 75% in arm A and 88.8% in arm B
Kim et al. [81]	2016	Prospective phase 2 study	32	Stage IV rectal cancer with synchronous liver metastases	25 Gy/5 fx	-	Surgical resection of rectum and liver accomplished in 25 patients (78%) R0 resection in 20 patients(63%) Rectal tumor downstaging in 54% of patients Median OS 38 months Median PFS 9 months
Bird et al. [80]	2017	Retrospective	78	Stage IV rectal cancer with synchronous liver metastases	50.4 Gy/28 fx (split course RT)	6.2 years	Radiological complete or partial response for local disease 90% Radiological complete or partial response for metastatic disease 66% Median OS in patients having unresectable metastatic disease at baseline 23 (19-28) months Estimated 3-year OS 62% for the 12 patients treated with radical surgery for both rectum and liver For patients not receiving rectal surgery, palliative re-irradiation or surgery at a later date needed in only 7%
Fossum et al. [91]	2017	Retrospective	93	Stage IV rectal cancer with synchronous distant metastases	Median 50.4 Gy/28 fx for LCRT Median 25 Gy/5 fx for SCRT (±intraoperative RT to median dose of 12.5 Gy)	43 (16-67) months	LR was not observed in patients receiving neoadjuvant RT 12 patients (26%) receiving no RT had LR Omission of neoadjuvant RT was associated with development of subsequent LR 5-year OS 43.3% for no RT group 5-year OS 58.3% for the RT group
Holliday et al. [76]	2017	Retrospective	38	Stage IV rectal cancer with synchronous distant metastases	25 Gy/5 fx	25 (14.75-42.25) months	1-year OS 97% 2-year OS 86.2% 3-year OS 76% 1-year PFS 52.1% 2-year PFS 22.7% 3-year PFS 17%
Bisschop et al. [79]	2017	Prospective, multicenter phase 2 study	50	Stage IV rectal cancer with synchronous distant metastases	25 Gy/5 fx	8.1 (6-9.8) years	Median OS 3.8 ( 0.5–9.4) years for all patients 2-year OS 74% 5-year OS 38% For 36 of 50 patients (72%) with radical (R0) primary tumor and metastatic site treatment, median OS 4.4 years, LR 5.6%, distant recurrence 80.6%
D'Hondt et al. [105]	2017	Retrospective multicenter study	18	Stage IV rectal cancer with synchronous liver metastases	-	20.5 (3.6–63.1) months	3 patients died and 10 patients had tumor progression during the follow-up period Median time to progression after liver surgery 4.2 (2.8–9.2) months
Labori et al. [84]	2017	Retrospective	45	Stage IV rectal cancer with synchronous liver metastases	50 Gy/25 fx (53%) or 25 Gy/5 fx (47%)	48 (6-85) months	Median OS 48.4 (43.3-54.8) months for all patients For the 40 patients completing the treatment sequence, median OS 49.7 (45.2-57.1) months and recurrence free survival 13 (16-30.3) months
Salvador-Rosés et al. [75]	2018	Prospective	35	Stage IV rectal cancer with synchronous liver metastases	50.4 Gy	38 (11-64) months	Median disease free survival 26 (23–28) months Median OS 53 (36-69) months for patients completing treatment strategy Median OS 25 (7-42) months for patients not completing treatment strategy
